# Histone Deacetylase Inhibitors Show a Potential Leishmanicidal Effect against Leishmania braziliensis in a Mouse Infection Model and Lead to Less Toxicity than Glucantime

**DOI:** 10.1021/acsomega.4c11381

**Published:** 2025-05-06

**Authors:** Luciana Ângelo de Souza, Lethícia Kelly Ramos Andrade, Joice de Melo Agripino, Victor Hugo Ferraz da Silva, Sabrina de Oliveira Emerick, Adriana Carneiro da Silva, Larissa Coelho Pereira, Graziela Domingues de Almeida Lima, Ingrid Rabite Garcia, Anna Cláudia Alves Souza, Tino Heimburg, Eduardo de Almeida Marques da Silva, Leandro Licursi de Oliveira, Luiz Otávio Guimarães Ervilha, Mariana Machado-Neves, Matheus Silva e Bastos, Raphael de Souza Vasconcellos, GustavoCosta Bressan, Abelardo Silva-Júnior, Raymond J. Pierce, Wolfgang Sippl, Juliana Lopes Rangel Fietto

**Affiliations:** † Departamento de Biologia Geral, 28120 Universidade Federal de Viçosa, Av. P. H. Rolfs s/n, Edifício Chotaro Shimoya, Viçosa, Minas Gerais CEP: 36570-900, Brazil; ‡ Departamento de Bioquímica e Biologia Molecular, Universidade Federal de Viçosa, Av. P. H. Rolfs s/n, CCBII, Viçosa, Minas Gerais CEP: 36570-900, Brazil; § Departamento de Veterinária, Universidade Federal de Viçosa, Av. P. H. Rolfs s/n, Viçosa, Minas Gerais CEP: 36570-900, Brazil; ∥ Institute of Pharmacy, 9176 Martin-Luther-University of Halle-Wittenberg, Kurt-Mothes-Str. 3, Halle (Saale) 06120, Germany; ⊥ Université de Lille, CNRS, Inserm, CHU Lille, Institut Pasteur de Lille, U1019—UMR 8204—CIIL—Centre d’Infection et d’Immunité de Lille, 1, Rue du Professeur Calmette, Lille 59000, France

## Abstract

Leishmania braziliensis is the primary cause of cutaneous leishmaniasis (CL) in the New World. Current treatments have significant limitations, including severe side effects and parasite resistance. Histone deacetylases (HDAC) are critical regulators of chromatin structure and represent potential drug targets for leishmaniasis. This study evaluated three HDAC inhibitors (HDACi), TH60, TH74, and TH85, in BALB/c mice infected with L. braziliensis, comparing their efficacy to the standard treatment, glucantime. Two doses were tested, and lesion size, parasite load, kidney and liver enzyme levels, and histopathological analyses were carried out. HDACi effectively reduced lesion size and parasite presence, with lower toxicity and fewer organ alterations than glucantime. Among the tested compounds, TH60 was the best-tested HDACi. These findings highlight the potential application of the tested HDACi as leishmanicidal agents against L. braziliensis, positioning them as promising candidates for developing new drugs targeting cutaneous leishmaniasis.

## Graphical abstract

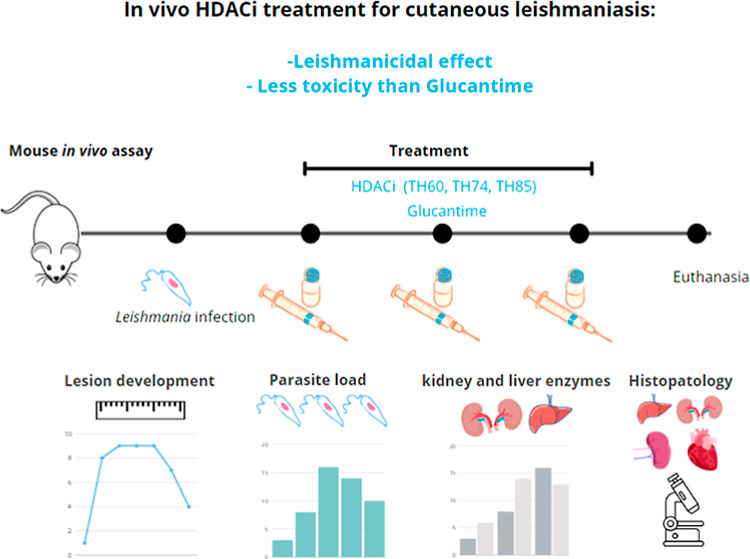

## Introduction

Leishmaniasis is a neglected tropical disease caused by over 20 species of Leishmania parasites, transmitted through the bite of infected sandflies of the genus Phlebotomus in the Old World and Lutzomyia in the New World.^1^ The disease cycle involves two parasite stages, promastigotes, which are introduced into the host during the sandfly bite, and amastigotes, which develop inside the phagolysosomes of host macrophages. Leishmania can manipulate macrophage signaling, such as inhibiting nitric oxide (NO) production, allowing amastigotes to proliferate and cause disease symptoms.^2^ As a result, amastigotes proliferate within these cells, leading to the clinical manifestations of leishmaniasis.^1^

Leishmaniasis primarily manifests as visceral leishmaniasis (VL) or American tegumentary leishmaniasis (ATL). VL, the most severe form, affects vital organs like the liver, spleen, and bone marrow, often fatal without treatment. ATL, the most common form, includes localized cutaneous leishmaniasis (CL), disseminated (DsCL), diffuse (DCL), and mucocutaneous leishmaniasis. Cutaneous leishmaniasis is endemic in regions like South America, North Africa, South Asia, and the Middle East. In South America, L. braziliensis is the most prevalent species causing ATL, notably in Brazil.^3^

Chemotherapy is a crucial measure and remains the principal form of treatment for leishmaniasis, as no licensed vaccine is currently available for the disease. However, vaccination strategies are under development. First-line drugs like meglumine antimoniate (glucantime) and sodium stibogluconate (pentostam) are often combined with second-line options such as amphotericin B, miltefosine, or methotrexate^4^ in the treatment of the disease, and they may lead to serious side effects, including cardiotoxicity,^5^ as well as liver and kidney injuries.^6^ Furthermore, the emergence of drug-resistant strains of Leishmania raises significant challenges in the treatment of leishmaniasis.^7^ Given this scenario, there is an urgent and critical need to search for new drugs with novel mechanisms of action to treat the disease.

Epigenetics is a rapidly expanding field of scientific research that deals with the mechanisms governing the structure and accessibility of DNA without altering its sequence and controlling vital biological phenomena like replication and transcription. Histone acetyltransferases (HAT) and deacetylases (HDAC), along with histone methyltransferases (HMT) and demethylases (HDM), are histone modifying enzymes (HME) that alter chromatin structure through post-translational modifications, like acetylation and deacetylation.^8^

The tight control of gene expression is fundamental for the balance of cell physiology, and disruption in this phenomenon orchestrated by HME is involved in the genesis and progression of several diseases, such as cancers^9^ and even COVID-19,^10^ which makes HMEs potential therapeutic targets.^8^

In humans, four main classes of HDAC are recognized and categorized by sequence similarity to yeast deacetylases, enzymatic activity, and subcellular localization. In this way, classes I, II, and IV are Zn_2_-dependent enzymes, and class III, also known as sirtuins, depend on NAD^+^ as a cofactor for their activity.^11^

In parasites, HDAC have been identified in Plasmodium falciparum,^12^ Trypanosoma brucei,^13^ Schistosoma mansoni^14^ and Leishmania, including L. braziliensis.^15^

The evolutionary relationship between humans and parasite HDAC has been described. Human HDAC are more closely related to those from Schistosoma species (such as S. mansoni). At the same time, they are more divergent from enzymes found in P. falciparum and trypanosomatids. Molecular modeling analyses of HDAC in these parasites revealed slight variations in the substrate binding site. This offers a promising starting point for selectively inhibiting kinetoplastid enzymes.^15^

“Drug repurposing” is a relevant approach that involves identifying new therapeutic applications for existing drugs already approved or undergoing clinical trials.^11^ In this way, several clinically validated HDAC inhibitors (HDACi) of structurally different classes, such as hydroxamic acids, short-chain fatty acids, cyclic tetrapeptides, and benzamides, have been tested against various diseases, such as breast cancer,^16^ leukemia,^17^ cardiac disease^18^ and brain cancer.^19^ In parasitic diseases, HDACi have long been employed.^20^ In leishmaniasis, studies have shown the potential leishmanicidal effect of HDACi against different Leishmania species, including Leishmania amazonensis,^21,22^ Leishmania donovani^21–25^ and Leishmania infantum.^25^ In a prior study, we evaluated an in-house library of hydroxamic acid derivatives as potential inhibitors of L. braziliensis HDAC. Five of the 78 compounds tested emerged as top candidates, exhibiting effective concentrations (EC_50_) ranging from 4.38 to 10.21 μM. These compounds also demonstrated selectivity indexes (SI) ranging from 6 to 21.7, which are important values as they align with the hit selection criteria established by the Drugs for Neglected Diseases Initiative (DNDi) outlined in.^26^ According to these criteria, a hit should exhibit a selectivity window greater than 10-fold to ensure sufficient differentiation between antiparasitic efficacy and cytotoxicity. The SI values observed in our study ensured a sufficient safety margin for in vivo testing, supporting their potential for further optimization and development as promising candidates for the treatment of L. braziliensis infections. Furthermore, these potential HDACi induced alterations in the cell cycle and triggered apoptosis in the parasite, indicating their potential as anti-parasitic agents. The compounds did not show significant toxicity on the host cell model macrophages and did not impact nitric oxide production in these cells.^27^

In the current study, we aimed to assess the impact of three previously identified HDACi (TH60, TH74, and TH85)^27^ in a mouse model of cutaneous leishmaniasis. We evaluated various parameters, including lesion size, parasite burden, biochemical markers, and tissue damage. Our findings align with the previous in vitro observations, indicating that these HDACi have promising potential for selectively targeting L. braziliensis, thereby offering a novel target for a therapeutic approach for cutaneous leishmaniasis. Overall, all these research studies strongly highlight the importance of epigenetic therapy through HDAC inhibitors in different diseases.

## Results

### Acute Toxicity Test

Acute toxicity testing is a preliminary method to assess the toxicity of pharmacological agents, since it evaluates the unwanted effects that occur soon after administering single or multiple doses of a test substance over a 24 h period.^28^ Hence, to ascertain the treatment doses against L. braziliensis in the animal infection model, TH74 was utilized for our toxicity assessment. The animals (*n* = 5) received the HDACi at concentrations of 5, 10 or 20 mg/kg intravenously for 2 weeks on alternate days. DMSO was used as the diluent negative control. The results were obtained following the completion of the six-dose regimen. As depicted in Table 1, the dose of 20 mg/kg proved lethal for one animal in the group in a few moments after the first application of TH74. Another death occurred minutes after the administration of the third dose at the same concentration. Following the application of the fourth dose at 20 mg/kg, two more animals in the group did not resist and died, also within a few minutes (Table 1).

**Table 1:** Results from the Acute Toxicity Test Show Lethality and Signs of Toxicity in BALB/c Mice after Administration of TH74^a^

dose (mg/kg/day/i.v.)	mortality	survival time (min)	signs of toxicity
control	0/5	>360	
DMSO	0/5	>360	
5	0/5	>360	
10	0/5	>360	
20	4/5	1, 3, 4, 4	anesthesia, ataxia, tremors and muscle spasms

a i.v.: intravenous; min: minutes.

Only one animal survived from the group that received a 20 mg/kg dose in the toxicity test. The observed signs of toxicity in these animals included anesthesia, ataxia, tremors, and muscle spasms. The control group (PBS only), along with the vehicle/diluent (DMSO) and the two other concentrations tested of TH74, showed no toxicity to the animals (Table 1). Therefore, the dosage of 20 mg/kg was excluded from subsequent tests, due to its notable toxic effects in the animals, as illustrated in Figure S2, depicting the mortality rate after treatments.

**Figure 1:**
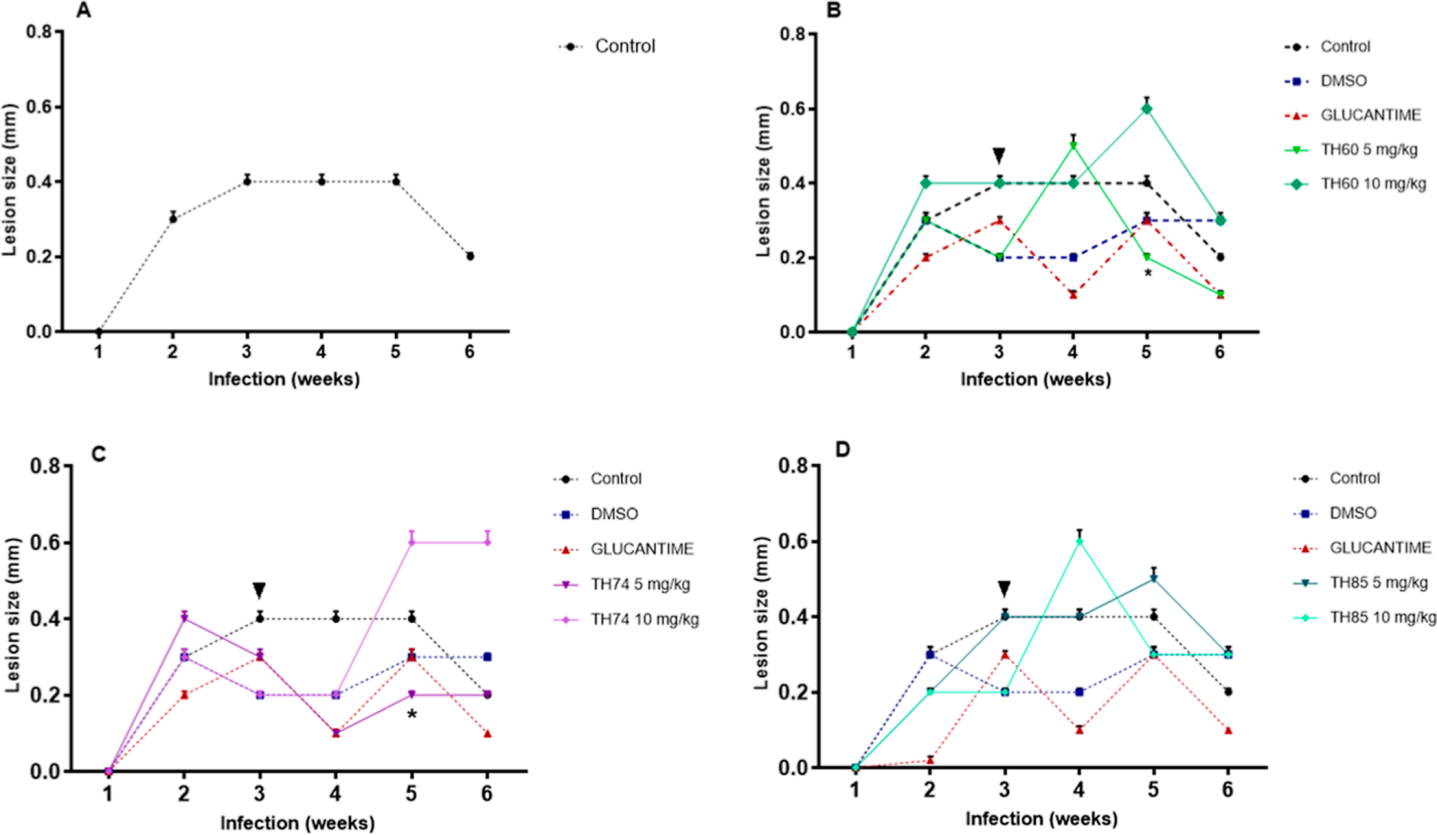
Lesion measurement in the footpad of BALB/c mice infected with L. braziliensis after treatment with TH60, TH74, and TH85. (A) Lesion size in the control. (B) Lesion size after treatment with TH60. (C) Lesion size after treatment with TH74. (D) Lesion size after treatment with TH85. The animals (*n* = 6) were treated with HDACi at concentrations of 5 or 10 mg/kg intravenously for 3 weeks on alternate days. Treatments began in the second week after infection (arrowhead) and were concluded in the fourth week after infection. Glucantime and DMSO were used as positive and negative/diluent controls, respectively. The control group received no treatment (PBS only). The results represent the mean and standard deviation of the thickness difference between the infected footpad (left) and the noninfected contralateral footpad after treatment. Measurements of lesion were made once a week. Data were subjected to unpaired Student’s *t*-test using GraphPad Prism version 5.03. Unpaired Student’s *t*-test is a statistical method used to compare the means of two independent groups to determine if there is a significant difference between them. It takes into account the alternative hypothesis, which assumes that there is a significant difference between the groups. The asterisk (*p < 0.0001) indicates a statistical difference between glucantime and TH60 (5 mg/kg) and between glucantime and TH74 (5 mg/kg).

The primary parameter used to assess the acute toxicity of a compound, reflecting the dose capable of causing mortality in 50% of the studied animal population, is the median lethal dose (LD_50_).^28^ Based on the preceding findings, the LD_50_ for TH74 was determined at 15.66 mg/kg (https://www.aatbio.com/tools/ld50-calculator) and can be seen in Figure S2 (Supporting Information).

### Treatment with HDACi

BALB/c mice, which are susceptible to Leishmania infection,^29^ were used to assess the effect of HDACi treatment. A pilot experiment with the specific strain of L. braziliensis MHOM/BR/75/M2904 was conducted to evaluate lesion development in the footpad and ear, as shown in Figures S1 and S3 (Supporting Information). This step had the goal of ensuring that the same strain used in the in vitro screening of TH compounds could be used in the in vivo assays. While the ear infection more closely resembles the kinetics of lesions in human cutaneous leishmaniasis, the footpad lesions developed more rapidly (detectable by the second week after infection) and were chosen for evaluating the efficacy of HDACi treatment in the L. braziliensis infection model. The successful establishment of infection in the ear, confirms its suitability for studies exploring treatment effects in different anatomical sites.

After standardizing the dynamics of lesion development, mice were infected with the stationary growth phase parasites and treated with the HDACi TH60, TH74, and TH85 at concentrations of 5 or 10 mg/kg intravenously via the tail vein from the second to the fourth week postinfection. The effect of the treatment with HDACi on mice’ footpad lesion is illustrated in Figure 1.

The lesions presented a nodular appearance without ulceration, and their size was determined by the difference in thickness between the infected footpad (left) and the noninfected contralateral footpad (control). In Figure 1A, the dynamics of the lesion in the control, where no treatment was applied, is shown. It can be observed that in the fifth week after infection, the lesion size decreased. As shown in Figure 1B,C for TH60 and TH74, respectively, in the fourth week after infection and the last week of treatment, the size of the lesion treated with 5 mg/kg of HDACi was smaller and statistically different from glucantime, the positive control. For TH85 (Figure 1D), however, in the same period (fourth week after infection) and in the same concentration (5 mg/kg), the lesion did not decrease, being larger than controls and other treatments. It is interesting to notice that in the fifth week after infection, when there was no treatment and the animals were euthanized, the size of the lesion decreased compared to the previous week after treatment with TH60 (5 mg/kg) (Figure 1B), and it was equal to that of mice treated with glucantime. Therefore, to better understand the treatment’s effects, we conducted analyses of parasite load.

### Evaluation of the Parasite Load in the Footpad

The quantification of parasite load in the infected footpad of mice may reflect the leishmanicidal effect of HDACi, corroborating the findings of lesion reduction as previously shown. Thus, the animals were euthanized in the fifth week after infection, and parasite load measurement was carried out. This was performed by limiting dilution, and Leishmania growth was monitored for at least 15 days, with the results shown in Figure 2.

**Figure 2:**
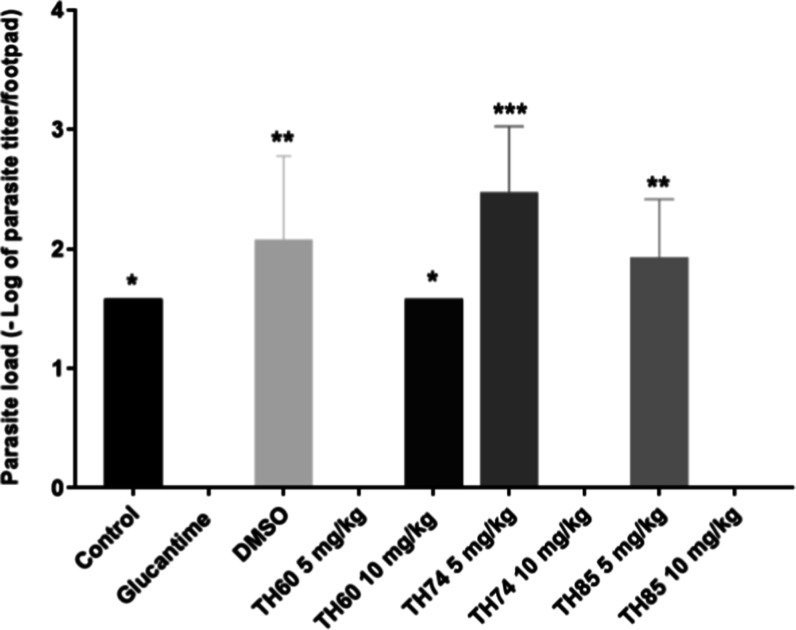
Parasite load in the footpad of BALB/c mice infected with L. braziliensis after treatment with TH60, TH74, and TH85. The animals (*n* = 6) were treated with HDACi at concentrations of 5 or 10 mg/kg intravenously for 3 weeks on alternate days. In the fifth week after infection, animals were euthanized, and the parasite load in the infected footpad was performed using the limiting dilution technique. Glucantime and DMSO were used as positive and negative/diluent controls, respectively. The control group received no treatment (PBS only). The results represent the mean and standard deviation of the −log parasite titer in the animals’ footpad. The absence of bars indicates that parasites were not detected in the infected footpad. Data were subjected to one-way ANOVA followed by Tukey’s test using GraphPad Prism version 5.03. One-way ANOVA followed by Tukey’s test is a statistical method used to compare the means of multiple groups to determine if there are any statistically significant differences between them. One-way ANOVA tests the overall differences among groups, while Tukey’s test is used for posthoc analysis to identify which specific groups differ. The asterisks (*p < 0.05) indicate statistical significance compared to glucantime. ***p = 0.0001; **p = 0.001; *p = 0.01.

It is possible to observe that Leishmania was detected in the control group and in those that received DMSO, as well as in the groups of animals treated with TH60 (10 mg/kg), TH74 (5 mg/kg) or TH85 (5 mg/kg). Parasites were not detected in groups treated with TH60 (5 mg/kg), TH74 (10 mg/kg) or TH85 (10 mg/kg).

### Dosage of Plasma Levels of Kidney Creatinine and Liver AST, ALT, ALP Enzymes

The determination of biochemical parameters in the blood of experimental animals is crucial, as it allows the assessment of potential metabolic changes, toxicity and tissue/organ function in response to infection, chemotherapy, or both.^30^ In this study, plasma levels of creatinine, ALT, AST, and ALP were evaluated after treatment with HDACi. Figure 3 illustrates the creatinine levels determined for the experimental groups.

**Figure 3:**
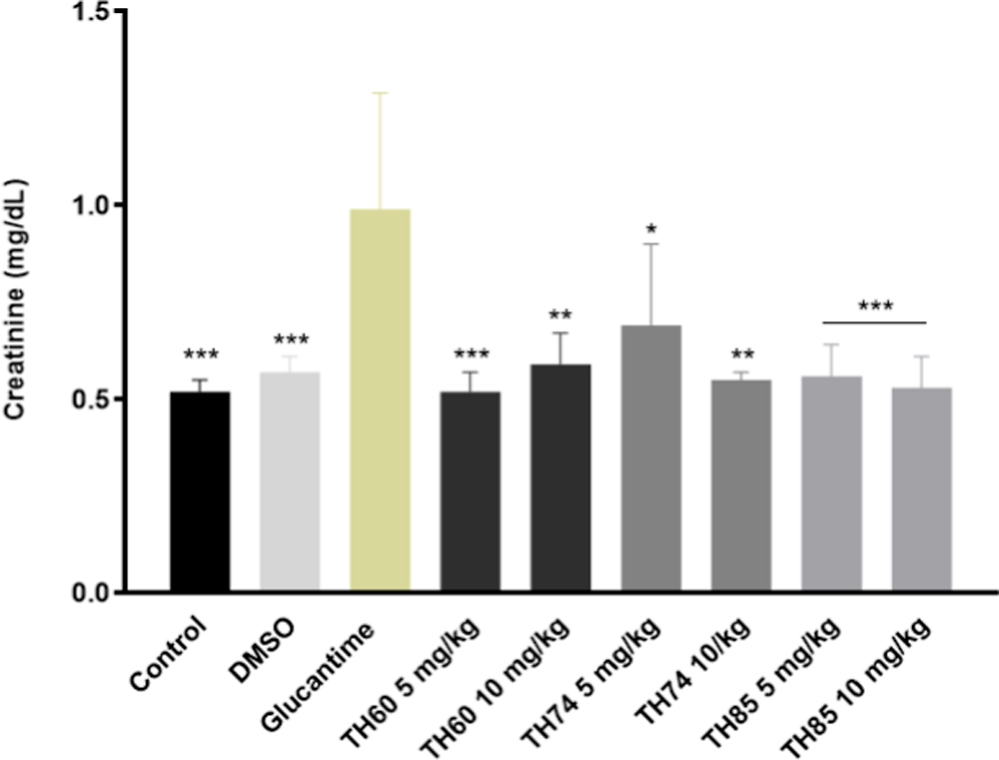
Dosage of plasmatic creatinine of BALB/c mice infected with L. braziliensis after treatment with TH60, TH74, and TH85. The animals (*n* = 6) were treated with HDACi at concentrations of 5 or 10 mg/kg intravenously for 3 weeks, on alternate days. In the fifth week after infection, animals were euthanized and prior to this procedure, the blood was collected to measure creatinine using a commercial kit. Glucantime and DMSO were used as positive and negative/diluent controls, respectively. The control group received no treatment (PBS only). The results are the mean and standard deviation of creatinine dosage in each group. Data were subjected to one-way ANOVA followed by Tukey’s test using GraphPad Prism version 5.03. One-way ANOVA followed by Tukey’s test is a statistical method used to compare the means of multiple groups to determine if there are any statistically significant differences between them. One-way ANOVA tests the overall differences among groups, while Tukey’s test is used for posthoc analysis to identify which specific groups differ. The asterisks (*p < 0.05) indicate statistical significance compared to glucantime. ***p = 0.0001; **p = 0.001; *p = 0.01.

The plasmatic creatinine levels were consistently lower across all treatments involving HDACi compared to glucantime (Figure 3). Notably, creatinine levels remained consistent between the control (0.52 ± 0.03 mg/dL) and TH60 (5 mg/kg) (0.52 ± 0.05 mg/dL). However, treatment with TH74 (5 mg/kg) (0.69 ± 0.21 mg/dL) showed the most significant increase in this biochemical parameter compared to all other treatments. A notable observation is the doubling of creatinine levels observed in the control and TH60 (5 mg/kg) treated mice compared to glucantime (control = 0.52 ± 0.03 mg/dL; TH60 = 0.52 ± 0.05 mg/dL, respectively) versus (glucantime = 0.99 ± 0.30 mg/dL) (Figure 3).

Alterations in liver function associated with a pathology or medication use, can be evaluated through the blood measurement of key markers such as AST, ALT and ALP.^6^ Figure 4 displays the plasma levels of these markers following treatment with HDACi or glucantime.

**Figure 4:**
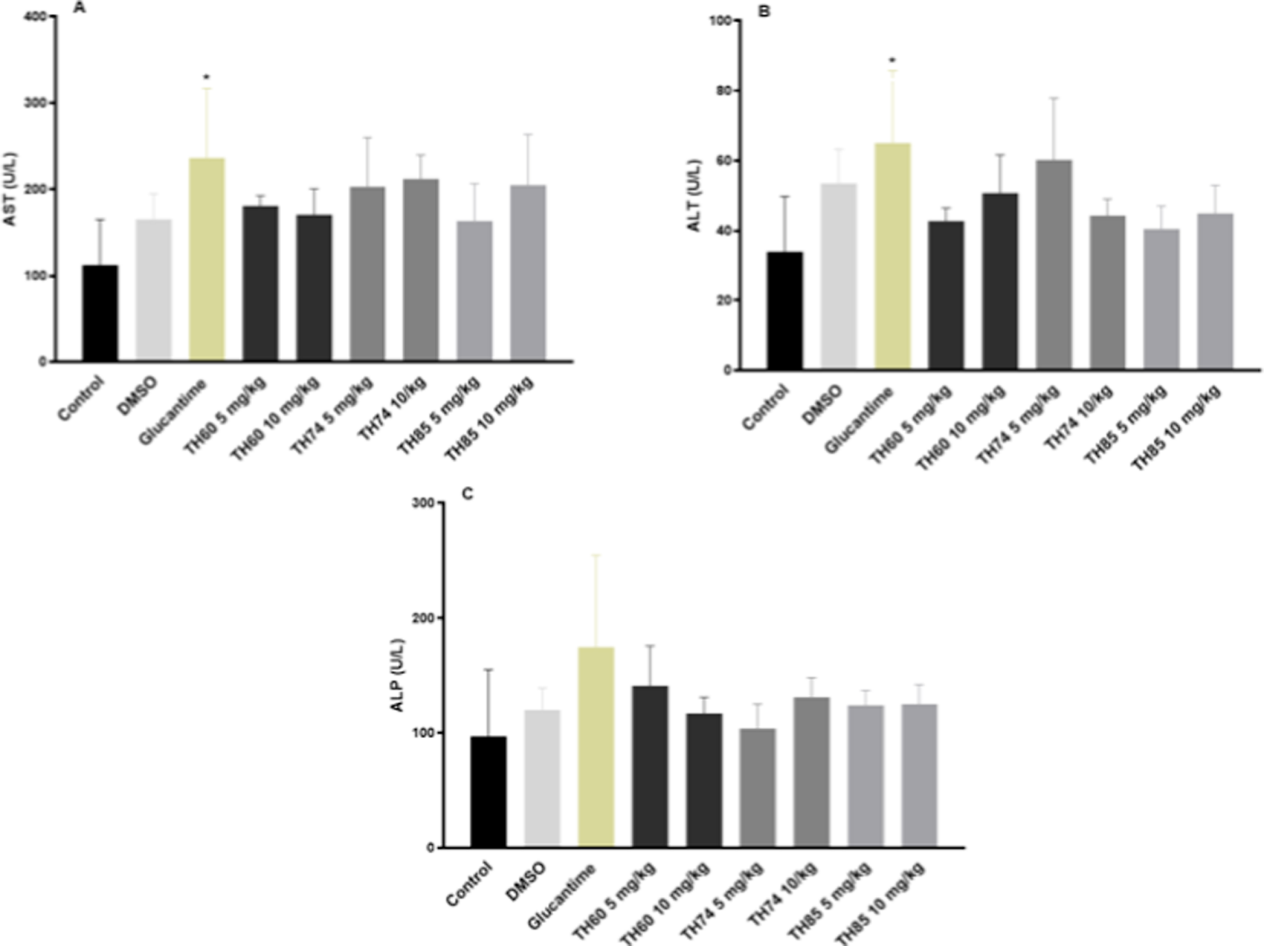
Dosage of plasma levels of liver enzymes AST, ALT and ALP of BALB/c mice infected with L. braziliensis after treatment with TH60, TH74, and TH85. (A) Plasma levels of AST. (B) Plasma levels of ALT. (C) Plasma levels of ALP. The animals (*n* = 6) were treated with HDACi at concentrations of 5 or 10 mg/kg intravenously for 3 weeks on alternate days. In the fifth week after infection, animals were euthanized, and prior to this procedure, the blood was collected to measure liver enzymes using commercial kits. Glucantime and DMSO were used as positive and negative/diluent controls, respectively. The control group received no treatment (PBS only). The results are the mean and standard deviation of the AST, ALT and ALP dosages in each group. Data were subjected to one-way ANOVA followed by Tukey’s test using GraphPad Prism version 5.03. One-way ANOVA followed by Tukey’s test is a statistical method used to compare the means of multiple groups to determine if there are any statistically significant differences between them. One-way ANOVA tests the overall differences among groups, while Tukey’s test is used for posthoc analysis to identify which specific groups differ. The asterisk (*p < 0.05) indicates statistical significance compared to control.

In Figure 4A, it is possible to observe that AST levels were lower after treatment with all HDACi compared to glucantime and were higher in relation to control and DMSO (with the exception of TH85 at 5 mg/kg relative to DMSO, which value was lower), although no statistically differences were found. However, when comparing control (112.50 ± 52.69 U/L) and glucantime (236.50 ± 80.83 U/L), AST levels are statistically higher in the latter. Moving to ALT levels (Figure 4B), most HDACi treatments exhibited lower marker levels, ranging from 40.50 ± 6.61 to 60.20 ± 17.64 U/L, compared to both glucantime (65 ± 20.75 U/L) and DMSO (53.50 ± 9.75 U/L), yet higher than the control (34 ± 15.75). No statistically significant differences were observed in these groups. Notably, TH74 (5 mg/kg) (60.20 ± 17.64 U/L) recorded higher values than control (34 ± 15.75) and DMSO (53.50 ± 9.75 U/L), but there are no statistical differences in these groups. Control group (34 ± 15.75) presented significantly lower ALT values in relation to glucantime (65 ± 20.75 U/L). Concerning ALP (Figure 4C), although no statistically significant differences were observed between control (97 ± 58 U/L), DMSO (120 ± 19 U/L) and HDACi treatments (104 ± 21 to 141 ± 35 U/L), a consistent decrease in this hepatic marker’s production was noted in all HDACi treatments (104 ± 21 to 141 U/L ± 35) relative to glucantime (175 ± 80 U/L).

### Histopathological Analyses of the Liver, Kidneys, Spleen, and Heart

To better evaluate the effects of the treatment with HDACi in the animals, we performed histopathological analyses of liver, kidney, spleen, and heart. Table 2 summarizes the obtained data. After treatments, animals from all experimental groups exhibited histological alterations in the liver, such as granulomas, inflammatory infiltrates, and congestion (Table 2).

**Table 2:** Histopathological Analyses of the Liver, Kidneys, Heart, and Spleen of BALB/c Mice Infected with L. braziliensis After Treatment with TH60, TH74 or TH85

	control (PBS)	GLUC	DMSO	TH60 (5 mg)	TH60 (10 mg)	TH74 (5 mg)	TH74 (10 mg)	TH85 (5 mg)	TH85 (10 mg)
Liver									
granulomas	+++	+	+++	++	+	+	+	+	+
granulomas/field	1.4 × 10^–1^ ± 0.11	3.3 × 10^–2^ ± 0.05	1.3 × 10^–1^ ± 0.08	5 × 10^–2^ ± 0.05	3.33 × 10^–2^ ± 0.05	3.33 × 10^–2^ ± 0.05	4 × 10^–2^ ± 0.05	3.33 × 10^–2^ ± 0.05	4 × 10^–2^ ± 0.05
inflammatory infiltrate	++	++	+++	++	++	++	+++	++	++
congestion	+++	+++	+++	++	++	++	++	++	+++
hemosiderin	+	+	+				+	+	+
Kidney									
congestion	++	++	++	+	++	+	++	+	+
Heart									
inflammatory infiltrate	+++	+++	+++	+	+	+	+	+	++
Spleen									
amastigotes inside macrophages	+		+						
megakaryocytes	++	++	++	+	+	+	+	+	+
hemosiderin	+++	+	+	+	++	+	++	+	+

The animals (*n* = 6) were treated with HDACi at concentrations of 5 or 10 mg/kg intravenously, for 3 weeks, on alternate days. In the fifth week after infection, animals were euthanized and organs were collected to histopathological analyses. Glucantime (GLUC) and DMSO were used as positive and negative/diluent controls, respectively. The control group received no treatment (PBS only). The results were expressed following the parameters for each histopathological pattern: (-) no histological alterations observed; (+) mild histological alterations; (++) moderate histological alterations; (+++) severe histological alterations. The number of granulomas was assessed in ten histological fields per animal, under a light microscope with 200× magnification.

It is possible to observe that liver alterations were more severe in control animals and those that received DMSO. On the other hand, the alterations were moderate in animals treated with TH60 (5 mg/kg). For animals treated with TH74 or TH85 at both concentrations of 5 or 10 mg/kg, and with TH60 (10 mg/kg), fewer granulomas were found. Regarding the presence of inflammatory infiltrates, these were moderate in animals treated with all HDACi, except for TH74 (10 mg/kg), where the quantity of these infiltrates was higher. The presence of congestion in the liver was also moderate in animals from groups treated with HDACi, except in those receiving treatment with TH85 (10 mg/kg). Hemossiderin was found in smaller amounts in control and DMSO groups and also in those treated with TH74 (10 mg/kg) or TH85 at both concentrations (5 or 10 mg/kg), not being detected in animals from other groups, TH60 (5 or 10 mg/kg) and TH74 (5 mg/kg). Comparing the HDACi treatments with the reference leishmanicidal drug glucantime, we can observe equal or reduced damage of organs in general.

Hemossiderin was found in the liver of animals from the control groups (PBS and DMSO) and also in those treated with TH74 (10 mg/kg) and TH85 (5 or 10 mg/kg).

Histopathological analysis showed renal congestion in animals from all groups, with more severe congestion in the control groups and those treated with TH60 and TH74 at 10 mg/kg.

Inflammatory infiltrates were observed in the hearts of animals from all experimental groups, with higher levels in the control groups (PBS, DMSO) and those treated with TH85 (10 mg/kg). In the spleen, amastigotes were found in macrophages from the control and DMSO groups. However, no amastigotes were detected in the spleen of HDACi-treated animals.

Megakaryocytes, which are platelet precursors, were found in the spleen of animals from all experimental groups, being more abundant in control groups (PBS and DMSO). Regarding hemosiderin, this was also detected in the spleen of animals from all experimental groups, being more abundant in those that received PBS (control) and those treated with TH60 or TH74 at 10 mg/kg concentration.

In relation to glucantime, it is observed that animals presented histological alterations of variable severity in all analyzed organs.

## Discussion

Animal models have long been used to elucidate mechanisms involved in human cutaneous leishmaniasis, including the types of immune cells involved, immune response profiles, signaling cascades for parasite elimination, and the search for new drugs.^31,32^ An acute toxicity test was initially conducted to determine treatment doses for infection. This test plays a key role in drug development by evaluating adverse effects occurring within 24 h after a single or multiple administrations of a test substance.^33^ The test was performed using the HDACi TH74 at concentrations of 20, 10 or 5 mg/kg/day. The pronounced toxicity observed for TH74 at 20 mg/kg may be attributed to the absence of the first-pass effect in intravenous administration. The drug bypasses the digestive tract and liver metabolism in this route, directly entering systemic circulation. This increases its bioavailability and leads to rapid toxic effects.^34^ Additionally, drug formulation (solution, suspension, emulsion) and stability at the time of administration are critical factors in i.v. delivery. In the case of a solution, precipitation can lead to pulmonary embolism.^35^ Precipitates were indeed observed in centrifuge tubes containing TH74 at 20 mg/kg, which may explain the high lethality in this group and indicate low compound solubility in phosphate-buffered saline.

Based on these results, the median lethal dose (LD_50_) for TH74 was determined to be 15.66 mg/kg. The LD_50_ is a primary parameter in evaluating acute toxicity.^36^ According to Berezovskaya’s classification, an intravenous LD_50_ between 0.7 and 40 mg/kg is categorized as highly toxic (Class 2). However, toxicity assessment also includes biochemical and histological parameters in animal models.^37^ Acute toxicity testing enabled the identification of safe doses (10 and 5 mg/kg) for treating L. braziliensis in mice, as no deaths occurred after the sixth treatment in these groups.

L. braziliensis is the main causative agent of cutaneous leishmaniasis in Brazil and is also widely distributed across Latin America, from Central America to northern Argentina.^38^ In addition to its epidemiological relevance, L. braziliensis has been extensively applied in screening studies for novel antileishmanial compounds. In BALB/c mice, L. braziliensis induces a distinct immune response compared to other Leishmania species. Unlike L. major, which triggers a strong Th2 response that leads to disease progression, L. braziliensis infection elicits a more balanced Th1/Th2 immune response that can control the infection and promote lesion resolution over time. The production of IFN-γ plays a key role in macrophage activation and parasite control. Thus, although BALB/c mice are susceptible, they do not necessarily develop chronic lesions.^31,39^

To ensure that the M2904 strain used in this study could lead to a classical model of infection to be used in the in vivo assays, we did a pilot experiment of ear (Figure S3) and footpad infection (Figure S1). The lesion in the footpad appeared as early as day 14 postinfection (second week), with a clear peak in lesion size observed at day 28, followed by self-healing as expected. This time point guided the therapeutic window; therefore, treatment with HDACi compounds was initiated in the second week postinfection. This is consistent with our observation that lesion size decreased in the control group by the fifth week postinfection, reflecting a characteristic self-healing process in BALB/c mice infected with this parasite species. In contrast, Figure S3 shows the lesion dynamics in the ear, where complete lesion development occurred only around day 49, with the healing process beginning approximately at day 56. These findings support the selection of the footpad as the infection site for in vivo evaluation, given the earlier and more measurable lesion development, which allows for better assessment of therapeutic efficacy.

Host immune responses, including the intrinsic self-healing capacity of BALB/c mice may influence variation in treatment efficacy across HDACi compounds. To better understand this, parasite burden was evaluated, revealing persistence of Leishmania in vehicle and control groups, as well as in certain HDACi-treated animals. For this study, our primary objective was to assess the effect of treatment on viable parasite burden, which is more directly correlated with therapeutic efficacy. The limiting dilution assay allowed us to achieve this goal by measuring the ability of parasites to proliferate after treatment. The persistence of parasites following treatment with TH60 (10 mg/kg), TH74 (5 mg/kg), and TH85 (5 mg/kg) could be due to the anti-inflammatory effects of HDACi, which may upregulate IL-10, IL-13, and IL-4, promoting alternative macrophage activation (M2 phenotype) and arginase activity, while downregulating nitric oxide synthesis, a key molecule in parasite killing.^40^ This suggests a dose-dependent and compound-specific immunological modulation by HDACi,^41^ reinforcing the importance of homology modeling for selective inhibitor design.^42^

The absence of detectable parasites in the TH60 (5 mg/kg) group also merits further investigation. The immune modulation triggered by HDACi might contribute to parasite control in specific treatment contexts. However, variability in intravenous drug administration via tail vein, affected by technique, absorption, and individual physiology, could influence serum concentrations and therapeutic efficacy, as highlighted in a PET imaging study by.^43^

Although the pharmacokinetics and immune markers were not the primary focus of this study, these aspects can be explored in future studies, including cytokine profiling and macrophage activation assays, to better understand the influence of HDACi on host responses.

In the in vivo assay, we observed that the vehicle (DMSO) appeared to promote lesion size reduction (Figure 1). It is important to acknowledge that DMSO is not an inert solvent. It possesses known immunomodulatory properties, including modulation of immune cell function and cytokine production. Depending on the context, DMSO can either enhance or suppress immune responses and is known to affect macrophages, T cells, and dendritic cells.^44^ Despite that, our data revealed the persistence of Leishmania in the vehicle group (DMSO), but not in many HDACi-treated groups, highlighting the specific action of TH compounds as leishmanicidal agents.

The determination of biochemical parameters in the blood of experimental animals is essential, as these parameters allow for the assessment of potential metabolic alterations as well as changes in tissue and organ function in response to an infection model, chemotherapy, or both.^45^ Plasma creatinine remained within the normal range for BALB/c mice (0.44 ± 0.11 mg/dL) following HDACi treatment (0.55–0.69 mg/dL), suggesting no renal toxicity. In contrast, glucantime-treated animals showed elevated creatinine levels (0.99 ± 0.30 mg/dL), suggesting higher nephrotoxicity. Creatinine is the primary biomarker of renal function and represents the final, irreversible product of creatine and phosphocreatine metabolism, particularly in skeletal muscle, where the concentration of these energy substrates is higher due to the tissue’s intrinsically high metabolic demand. As creatinine is produced at a relatively constant rate, it must be efficiently eliminated from the body to avoid toxicity. In healthy individuals, this elimination is carried out by the kidneys.^46–48^

Alterations in liver function, whether due to underlying pathology or drug administration, can be assessed through the measurement of key blood biomarkers such as aspartate aminotransferase (AST), alanine aminotransferase (ALT), and alkaline phosphatase (ALP).^49^ Literature data for liver enzymes in mice are limited, but Spinelli et al. reported ALT (44.33 ± 4.78 U/L) and AST (21.66 ± 12.60 U/L), while ALP levels range between 210.43 and 323.57 U/L.^50^ Although some values in our study exceeded baseline levels, they did not approach the thresholds typically associated with hepatocellular damage (10–100× above normal). All HDACi treatments showed lower AST, ALT, and ALP levels than glucantime, indicating lower hepatotoxicity.

To better evaluate the effects of HDACi treatment in the animals, histopathological analyses of the liver, kidney, spleen, and heart were performed. The liver is responsible for metabolizing drugs^51^ and can be affected by infectious processes such as visceral leishmaniasis.^52^ Visceralization by dermotropic Leishmania species distant from the site of infection can also occur and has been reported in mice, being related to the parasite density used in the inoculum for experimental infections.^30,53^ In humans, visceralization by dermotropic species is rare but has been reported in immunosuppressed patients.^54^ Liver-resident macrophages, known as Kupffer cells, are the first line of defense against Leishmania and can form granulomas, clusters of macrophages attempting to contain the parasite.^55,56^ Thus, in the PBS (control) and DMSO groups, it is possible that visceralization occurred due to the high parasite load resulting from the absence of treatment, which may explain the greater number of granulomas observed in these groups. In contrast, animals treated with HDACi showed fewer granulomas in the liver, possibly due to reduced hepatic parasite load following treatment.

Regarding the moderate to high levels of inflammatory infiltrates in the livers of treated animals, these could be associated with granuloma formation or might reflect liver damage caused by the HDACi themselves.^57^ In terms of hepatic congestion, its more severe occurrence in animals from the PBS (control), DMSO, and TH85 (10 mg/kg) groups may be related to heart damage caused either by the treatment or as a consequence of L. braziliensis infection, as these groups presented higher levels of inflammatory infiltrates in cardiac tissue. Cardiac dysfunction is a known cause of hepatic congestion, as described by.^58^

Hemosiderin was observed in the livers of animals from the control groups (PBS and DMSO), as well as in those treated with TH74 (10 mg/kg) and TH85 (5 or 10 mg/kg). This may be due to red blood cell destruction as a consequence of congestion.^58^

The kidneys are involved in the excretion of metabolic waste products and in various regulatory processes, such as blood pressure control.^59^ Histopathological analyses showed renal congestion in animals from the control groups (PBS and DMSO) as well as those treated with HDACi. However, the severity of this congestion was higher in the control groups and in animals treated with TH60 and TH74 at 10 mg/kg. As with hepatic congestion, renal congestion may result from heart dysfunction and is also associated with abnormal blood return to the heart via major veins.^60^

Inflammatory infiltrates in the heart were detected in animals from all experimental groups, with greater severity in the control groups (PBS and DMSO) and in those treated with TH85 (10 mg/kg). Myocarditis, an inflammation of the heart muscle, can compromise the heart’s pumping ability.^61^ It has been described in dogs as a cardiologic alteration resulting from the systemic response triggered by Leishmania infection.^62^ Data on cardiac involvement in human leishmaniasis are rare.^63^ Therefore, the inflammatory infiltrates observed in the hearts of the animals may be related to the systemic immune response against infection rather than being caused by HDACi treatment. Furthermore, the potential use of HDACi in treating cardiovascular conditions has been proposed,^64^ which further supports the idea that the cardiac damage observed may be infection-related rather than treatment-induced.

Visceralization of the spleen, as with the liver, can also occur during Leishmania infection.^54,65^ The presence of amastigotes inside macrophages in animals from the PBS (control) and DMSO groups reinforces this possibility, as these groups did not receive any treatment. In contrast, no amastigotes were detected in the spleens of animals treated with HDACi, suggesting a possible efficacy of these inhibitors in controlling infection in this organ.

Megakaryocytes are platelet precursors and were observed in the spleens of animals from all experimental groups, but were more abundant in the control groups (PBS and DMSO). Their presence indicates hematopoietic activity,^65^ which can be altered by Leishmania infection.^66^ Thus, the presence of amastigotes in the spleens of the control groups may have had a greater influence on hematopoiesis in these animals. Hemosiderin was also found in the spleens of all groups, being more abundant in those that received PBS (control) and in those treated with TH60 or TH74 at 10 mg/kg. It is not possible to rule out the possibility that the infection itself caused other splenic alterations not evaluated in this study, which may have led to increased hemosiderin accumulation.^67^

Regarding treatment with glucantime, animals exhibited histological alterations of varying severity in all organs analyzed, liver, kidneys, heart, and spleen. The toxicity of this pentavalent antimonial is well documented in the literature.^68,69^ The proposed mechanism for this toxicity involves the accumulation of the drug in its trivalent form Sb­(III) in the organs, leading to intracellular glutathione depletion, inhibition of glutathione reductase, and increased oxidative stress in tissues.^68^ Furthermore, the previously presented data on renal and hepatic biomarkers support the histopathological findings, highlighting the higher toxicity of glucantime compared to HDACi.

The histopathological results demonstrate that treatment of L. braziliensis infection with HDACi caused equal or less organ damage than that observed in the control groups (PBS, DMSO, and glucantime), suggesting lower toxicity of these inhibitors compared to a drug currently used in the treatment of leishmaniasis. Additionally, the similarities in tissue alterations found in the PBS and DMSO groups indicate that the effects observed are likely due to the infection itself rather than DMSO toxicity, reinforcing the safety of DMSO as a vehicle in experimental treatments.

In this study, we intended to explore the three previously selected HDAC inhibitors in an in vivo assay for the first time. It is essential to highlight that this initial exploratory study provides valuable data for future optimization of these compounds, including dose adjustments, further pharmacokinetic assessments, and chemical structural improvements. Our findings suggest that TH60, TH74, and TH85 exhibited distinct in vivo effects, reinforcing the value of testing multiple compounds rather than focusing solely on one compound. In spite of the fact that the TH compounds led to moderate effects compared to the reference drug glucantime, this broader evaluation helps refine the selection of new lead compounds and supports more informed decisions in subsequent studies, aligning with the urgent need to identify new compounds for the treatment of leishmaniasis.

## Conclusion

Treatment of footpad lesions in BALB/c mice with HDACi after infection with L. braziliensis showed that TH60 (5 mg/kg) was the most effective HDACi in reducing lesions, even with greater efficacy than that of glucantime. The lesion reduction by TH60 correlates with the absence of parasites in the lesion. TH74 (10 mg/kg) also effectively reduced the lesion, equaling glucantime, and Leishmania was not detected in the lesion after the treatment. All three HDACi (TH60, TH74, and TH85) at the tested concentrations showed lower renal and hepatic toxicity than glucantime in plasma levels of the analyzed markers, creatinine, AST, ALT, and ALP. Histopathological analyses showed that treatment with HDACi led to fewer alterations in the organs of animals compared to glucantime, with HDACi being as effective as the mentioned medication in controlling infection in the liver and spleen. Cardiac toxicity was lower for HDACi compared to glucantime. Furthermore, the animals did not show variation in the normal weight or behavior after infection and treatment with HDACi.

Overall, the data from this study demonstrate the potential application of the tested HDACi as leishmanicidal agents against L. braziliensis and highlight them as promising compounds for the development of new drugs for the treatment of cutaneous leishmaniasis.

## Materials and Methods

### Parasites

L. braziliensis MHOM/BR/75/M2904 promastigotes were cultivated in Grace’s medium (Grace’s Insect Medium, Gibco, CA, USA) supplemented with 10% inactivated fetal calf serum (LGC Biotecnologia, SP, Brazil), l-glutamine (2 mM) (Serva Electrophoresis & Life Science Products, NY, USA) and penicillin (100 μg/mL) (USB Corporation, OH, USA), pH 6.5 at 25 °C in BOD chamber, as described by.^27^

### Animals

Female BALB/c mice aged 6–8 weeks (weight, 20–23 g), sourced from the Central Animal Facility of the Federal University of Viçosa—UFV, were utilized in the experiments. Animals were housed in the animal holding facility of the Departamento de Bioquímica e Biologia Molecular (DBB) of the UFV, and they were kept in each ventilated polycarbonate cage under specific pathogen-free conditions, with a controlled temperature of 25 ± 2 °C, 12 h light/dark cycles and relative humidity (60–70%). Animals received food and water ad libitum. Animals were provided with a seven-day acclimatization period before conducting the experiments. The experimental protocols were approved by the Ethics Committee of Animal Use of the Universidade Federal de Viçosa (CEUA/UFV, protocol 52/2017).

### Culturing Metacyclic Promastigotes Forms of L. braziliensis

Metacyclic promastigotes forms of L. braziliensis were obtained by spiking 10^5^ parasites/mL into a culture bottle. These parasites were cultured in supplemented Grace’s medium as described in ref 27 for 7 days until the stationary growth phase was reached. Metacyclic promastigotes in subculture P3 were used in animal infection models. The parasites were passed through BALB/c mice to maintain infectivity before subculturing P10. While we did not perform a specific enrichment step for metacyclic forms, the use of stationary-phase promastigotes is a well-established method for obtaining infective parasites for in vivo studies^70^

### Drug Compounds

The HDACi TH60, TH74, and TH85 were synthesized and purified as described before^71^ and were utilized to treat animal infection. These compounds were chosen from the top 5 identified in our prior study.^27^ The lyophilized compounds were first diluted in sterile dimethyl sulfoxide (DMSO) (Neon Comercial Reagentes Analíticos Ltd.a, SP, Brazil) (vehicle) at a maximum concentration of 1% (w/v), and subsequently in sterile PBS (v/v), pH 7.4, to achieve the desired concentrations (as described below). These preparations were conducted in a biological safety cabinet to avoid contamination and 1 day before the experiments. Compounds were then stored at −20 °C until used for animal treatment.

### Acute Toxicity Test

Following the acclimatization period, the animals were randomly allocated into five groups (*n* = 5) and subjected to an acute toxicity test involving the administration of TH74 and DMSO. The HDACi and DMSO were administered intravenously into the tail of mice for a 2 week period on alternate days, employing a 31-gauge needle (G) (6 mm × 0.25 mm). The concentrations and duration of administration were based on recommendations from the Ministério da Saúde, the Brazilian Ministry of Health, for treating cutaneous leishmaniasis caused by L. braziliensis, as well by studies conducted with the same species in an animal infection model.^72–75^

The groups were as follows: group 1—control, received PBS (i.v.) and therefore, no treatment; group 2, the negative control, received DMSO (1% v/v; i.v.). This was the same concentration used to dilute the compounds; group 3, received HDACi TH74 (20 mg/kg/day/i.v.); group 4 received HDACi TH74 (10 mg/kg/day/i.v.) and group 5 received HDACi TH74 (5 mg/kg/day/i.v.). TH74 was selected for the acute toxicity test because it represents one of the most promising HDAC inhibitors (HDACi) within the TH class of compounds. It has demonstrated significant activity against intracellular amastigotes of L. braziliensis in vitro.^27^

### Dynamics of Lesion Development in Footpad and Ear

Following the acclimatized period, the animals were randomly allocated into two groups (*n* = 6) and subjected to the development of footpad and ear lesions. The groups were as follows: group 1—control, noninfected; group 2, infected with L. braziliensis promastigotes (described previously). Infections were performed using a 31 G needle.

To assess lesion development, animals were infected subcutaneously in the left hind footpad with 10^7^ parasites in 40 μL of PBS. Measurements were taken starting from the second week of infection, continuing weekly for 9 weeks. Body weight and lesion size were monitored, with lesion size determined by the difference in thickness between the infected and uninfected footpads using a micrometer (model 1015 MA; LS, Starret Co, Itu, SP, Brazil). For ear lesions, animals were infected intradermally in the left ear with 10^^^5 parasites in 10 μL of PBS. Lesions were measured weekly for 4 weeks using a digital Vernier caliper (Kahakiboy, Shenzhen, China), and body weight was also recorded.

### Animal Infection in the Footpad with L. braziliensis

Following the acclimatized period, the animals were randomly allocated into nine groups (*n* = 6) and subjected to the development of footpad lesions, as previously described. After 7 days of infection or at the onset of the second week of infection, and then once a week for 4 weeks after that (from the beginning of the second week until the end of the fifth week after infection), body weight and the size of the lesion were measured. A micrometer (model 1015 MA; LS, Starret Co, Itu, SP, Brazil) was used to measure the lesion size, determined as previously described.

### Treatment of Lesion in the Footpad with HDACi

The treatment protocol involving TH60, TH74, TH85, and other tests was conducted in the animal’s postfootpad infection (described above). At the onset of the third week of infection (or in the second week after infection), body weight was measured and the compounds were administrated i.v. in the tail of the mice using a 31G needle. All treatments were in a volume of 200 μL as in the untreated control (group 1) and lasted for 3 weeks (from the beginning of the second week until the end of the fifth week after infection), with administration occurring on alternate days.

The glucantime used in the experiments was kindly provided by the René Rachou Institute—Oswaldo Cruz Foundation (FIOCRUZ MINAS), Belo Horizonte, Brazil. All HDACi and controls were prepared in a biological safety cabinet to avoid contamination. The administered doses of HDACi and controls were established after the acute toxicity test (described above) and based on recommendations from the Ministério da Saúde for treating cutaneous leishmaniasis caused by L. braziliensis.^72^

The groups were as follows: group 1—control, received PBS (i.v.) and therefore, no treatment; group 2, the negative control, received DMSO (1% v/v; i.v.). This was the same concentration used to dilute the compounds; group 3, positive control, treated with glucantime (20 mg/kg/day/i.v.); group 4, treated with TH60 (5 mg/kg/day/i.v.); group 5, treated with TH60 (10 mg/kg/day/i.v.); group 6, treated with TH74 (5 mg/kg/day/i.v.); group 7, treated with TH74 (10 mg/kg/day/i.v.); group 8, treated with TH85 (5 mg/kg/day/i.v.); group 9, treated with TH85 (10 mg/kg/day/i.v.).

### Euthanasia

At the onset of the sixth week of infection (or at the fifth week after infection), mice were weighed and euthanasia was performed using the cervical dislocation technique, replacing the use of barbiturates or other injectable general anesthetics recommended by Resolution 714 of the Federal Council of Veterinary Medicine (CFMV) in Brazil, since the use of anesthetics can interfere with the immune response of the animals.^76^

### Evaluation of the Parasite Load in the Footpad

The number of Leishmania in the infected footpad of the animals was estimated by the limiting dilution test, as described previously.^77^ Briefly, the infected left footpad was harvested and weighed. Then, footpads were homogenized in a tissue grinder and resuspended in supplemented Grace’s medium (previously described). The cell suspension was submitted to 5-fold serial dilutions and after 15 days, the presence of Leishmania was evaluated using an inverted microscope (Leica Microsystems, Wetzlar, Germany). The parasite load was calculated and expressed as −logarithm (−log) considering the last dilution where the parasite was detected. Protocol experimentation was adapted from.^78^

### Dosage of Plasmatic Levels of Kidney and Liver Enzymes

Biochemical assays were performed on plasma samples from the animals. Prior to blood collection, 200 μL of sodium heparin (2.5 U); (Hepamax-S, Blau Farmacêutica, Cotia, São Paulo, Brazil) diluted in 0.9% (w/v) NaCl saline solution, were administrated intravenously in mice. After 1 h, blood was collected and plasma stored at −20 °C for later biochemical analysis. Commercial kits from Bioclin (Quibasa-Bioclin, Belo Horizonte, Brazil) were used to measure renal marker creatinine and liver markers: pyruvic transaminase (ALT), oxaloacetic transaminase (AST), and alkaline phosphatase (ALP). The kits were provided through the Bioclin Educar Project. Biochemical analyses were conducted in collaboration with the Laboratory of Clinical Analysis at the Federal University of Viçosa.

### Histopathological Analyzes

Liver, kidneys, spleen, and heart were collected after euthanasia for histopathological analysis. The organs were weighed, fixed in Karnovsky solution^79^ for 24 h, dehydrated in ethanol, clarified in xylene, and embedded in paraffin. Semiserial sections (5 μm thick) were obtained, stained with hematoxylin–eosin (HE), and mounted with Entellan. Ten regions per organ were examined using a light microscope at 400× magnification. Histopathological alterations were evaluated semiquantitatively, with four damage levels: (-) no alterations, and, therefore, with the maintenance of the organ’s normal tissue architecture; (+) discrete, which involves the occurrence of alteration in one of the histological fields analyzed, in at least one animal per group; (++) moderate, when the pathology was found in two or more histological fields analyzed, in at least one animal per group and (+++) severe, when the histological alteration was found in more than two histological fields, in two or more animals per group, based on.^80^ Amastigotes of L. braziliensis in the spleen were observed at 1000× magnification, and the number of granulomas was assessed at 200× magnification. Histological analysis was conducted in collaboration with Professor Mariana Neves from the Graduate Program in Cellular and Structural Biology at UFV.

### Statistical Analysis

All numeric data are shown as the means ± standard deviation. Statistical analyses were carried out by one-way ANOVA and unpaired Student’s *t*-test. When *p* < 0.05, the results were considered statistically significant and were identified by an asterisk (*). LD_50_ dose was calculated by the online AAT Bioquest Inc. Quest graph LD_50_/ED_50_ calculator.^81^ Microsoft Excel (Microsoft Office Software System) and GraphPad Prism 5.03 (GraphPad Software Inc.) were used to perform the analyses.

## Supplementary Material



## Notes

Pilot experiment dynamics of lesion in the footpad and in the ear and percentage of mortality (PDF)

## #

Universidade Federal de Alfenas, Minas Gerais, 37130-001, Brazil

## ¶

Matheus Silva e Bastos: Department of Pharmacology & Toxicology, Indiana University School of Medicine, 635 Barnhill Drive, MS-A405B Indianapolis, IN 46202, United States.

## ∇

Universidade Federal de Alagoas, Alagoas, 57072-970, Brazil.

## Abbreviations

ALP: alkaline phosphatase
ALT: pyruvic transaminase
AST: oxaloacetic transaminase
A-ParaDDisE: anti-parasitic drug discovery in epigenetics
ATL: American tegumentary leishmaniasis
CL: cutaneous leishmaniasis
DCL: diffuse cutaneous leishmaniasis
DsCL: disseminated cutaneous leishmaniasis
LD_50_: lethal dose
ML: mucocutaneous leishmaniasis
DC: dendritic cells
DMSO: dimethyl sulfoxide
HAT: histone acetyltransferases
HDAC: histone deacetylases
HDACi: HDAC inhibitors
HME: histone modifying enzymes
HMT: histone methyltransferases
HDM: histone demethylases
HME: histone modifying enzymes
i.v.: intravenous or intravenously
NO: nitric oxide
PBS: phosphate-buffered saline
ROS: reactive oxygen species
SAHA: suberoylanilide hydroxamic acid
