# Supplementary material for: Histone Deacetylase Inhibitors Show a Potential Leishmanicidal Effect against Leishmania braziliensis in a Mouse Infection Model and Lead to Less Toxicity than Glucantime
Source: ACS Omega. 2025 May 6;10(19):19466–79. doi: 10.1021/acsomega.4c11381 (PMC12096192; doi:10.1021/acsomega.4c11381)
Supplement: Supplementary file 1 [file ao4c11381_si_001.pdf]

Histone deacetylase inhibitors show a potential leishmanicidal effect against *Leishmania braziliensis* in a mouse infection model and lead to less toxicity than Glucantime

Running title: HDAC inhibitors against *Leishmania braziliensis* in infected mice

Authors: Luciana Ângelo de Souza[1,2], Lethícia Kelly Ramos Andrade[2], Joice de Melo Agripino[2], Victor Hugo Ferraz da Silva[1], Sabrina de Oliveira Emerick[1], Adriana Carneiro da Silva[1], Larissa Coelho Pereira[2], Graziela Domingues de Almeida Lima[1a], Ingrid Rabite Garcia[1], Anna Cláudia Alves Souza[2], Tino Heimburg[4], Eduardo de Almeida Marques da Silva[1], Leandro Licursi de Oliveira[1], Luiz Otávio Guimarães Ervilha[1], Mariana Machado-Neves[1], Matheus Silva e Bastos[2,b], Raphael de Souza Vasconcellos[2], Gustavo Costa Bressan[2], Abelardo Silva-Júnior[3c], Raymond J. Pierce[5], Wolfgang Sippl[4] and Juliana Lopes Rangel Fietto[2,*]

* jufietto@ufv.br

Affiliations:

[1]Departamento de Biologia Geral, Universidade Federal de Viçosa, Av. P. H. Rolfs s/n, Edifício Chotaro Shimoya, Viçosa, Minas Gerais, CEP: 36570-900, Brazil

[2]Departamento de Bioquímica e Biologia Molecular, Universidade Federal de Viçosa, Av. P. H. Rolfs s/n, CCBII, Viçosa, Minas Gerais, CEP: 36570-900, Brazil

[3]Departamento de Veterinária, Universidade Federal de Viçosa, Av. P. H. Rolfs s/n, Viçosa, Minas Gerais, CEP: 36570-900, Brazil

[4]Institute of Pharmacy, Martin-Luther-University of Halle-Wittenberg, Kurt-Mothes-Str. 3, 06120 Halle (Saale), Germany.

[5]Université de Lille, CNRS, Inserm, CHU Lille, Institut Pasteur de Lille, U1019 - UMR 8204 - CIIL - Centre d'Infection et d'Immunité de Lille, 1, Rue du Professeur Calmette, 59000 Lille, France.

***Corresponding author:** Juliana Lopes Rangel Fietto - Phone (+55) 31 3612-2465. Departamento de Bioquímica e Biologia Molecular – Universidade Federal de Viçosa, Av. P. H. Rolfs s/n, **CCBII**, Viçosa, Minas Gerais, CEP: 36570-900, Brazil. E-mail: jufietto@ufv.br

Supporting Information

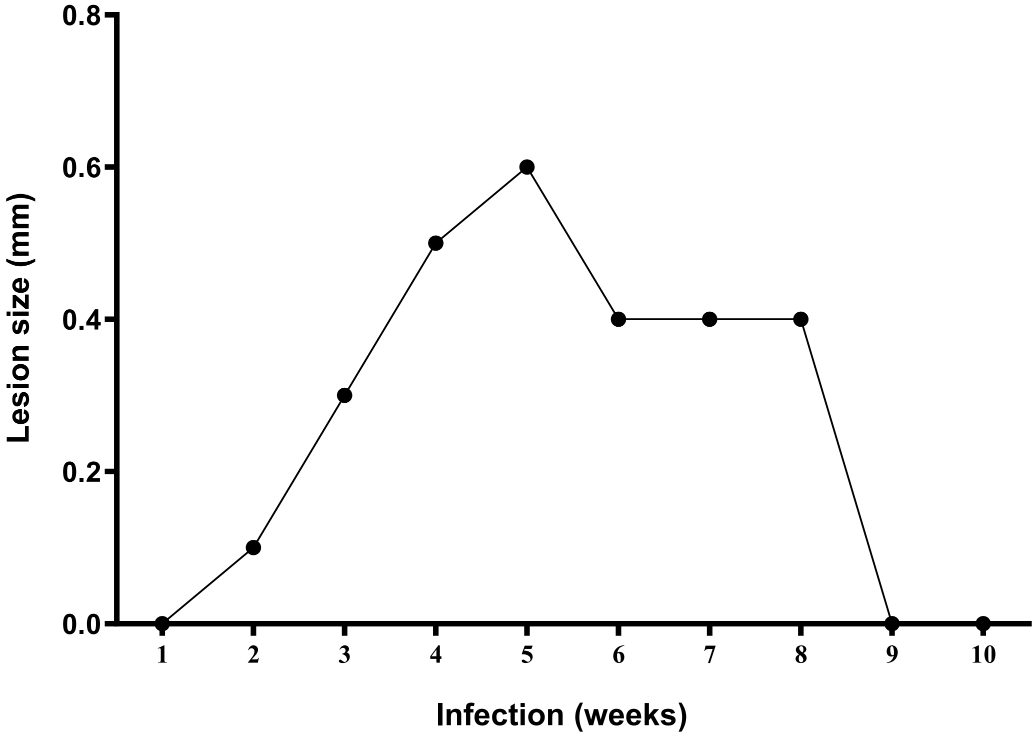

Figure S1. **Pilot experiment dynamics of lesion in the footpad of BALB/c mice infected with *L. braziliensis*.** The animals (n = 6) were infected subcutaneously in the plantar pad of the left hind footpad with $1x10^7$ promastigotes in stationary growth phase and the lesion was measured for nine weeks using a micrometer. The results reflect the mean and standard deviation of the difference in thickness between the infected footpad (left) and the contralateral uninfected footpad.

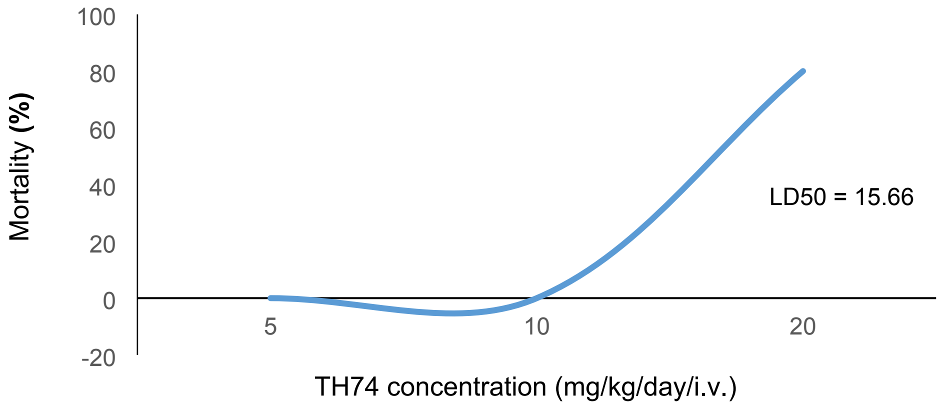

Figure S2. Percentage (%) of mortality plotted against TH74 concentrations in the acute toxicity test. The animals (n = 5) received the HDACi at concentrations of 5 mg/kg, 10 mg/kg or 20 mg/kg intravenously for two weeks on alternate days. The results depict the mortality of animals in each group at the end of the six doses application. Lethal dose (LD50) was calculated using the online AAT Bioquest Inc. Quest graph LD50/ED50 calculator [29]. LD50 reflects the dose of a substance required to kill 50% of the population within a specified period. Microsoft Excel (Microsoft Office Software System) was used to perform graphical representation.

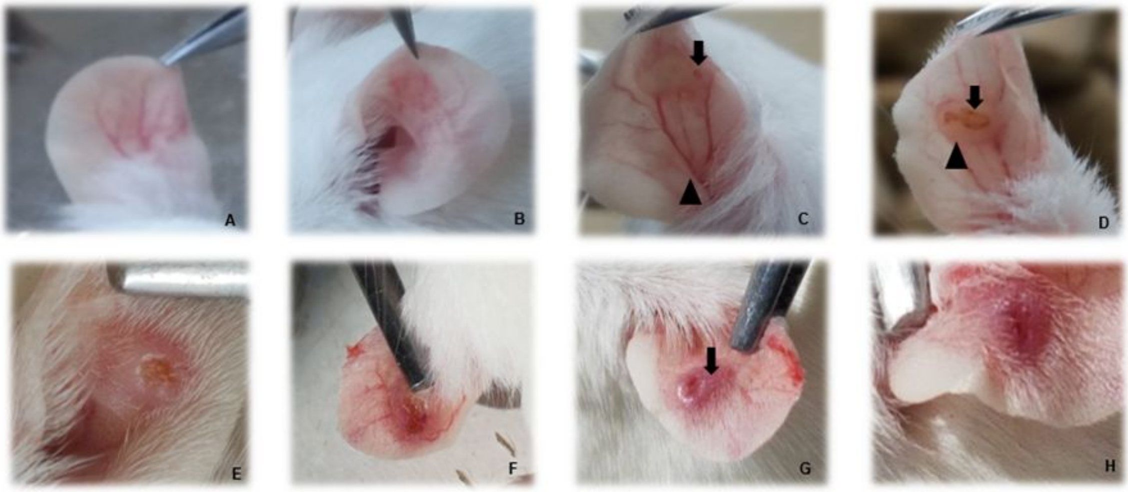

Figure S3. Pilot experiment of lesion dynamics in the ear of BALB/c mice infected with *L. braziliensis*. The animals (n = 6) were intradermally infected in the central left auricular pavilion with $1x10^5$ stationary-phase promastigotes, and the size and characterization of the lesion were determined over eight weeks using a digital caliper and the scoring method developed by (SCHUSTER et al., 2014). Results are shown in days after infection. A) PP 15 days; B) PA 28 days; C) PP 28 days; D) PP 35 days; E) PA 35 days; F) PP 49 days; G) PA 49 days; H) PA 56 days. PA = anterior portion; PP = posterior portion.
